# The Phosphoarginine Phosphatase PtpB from *Staphylococcus aureus* Is Involved in Bacterial Stress Adaptation during Infection

**DOI:** 10.3390/cells10030645

**Published:** 2021-03-14

**Authors:** Mohamed Ibrahem Elhawy, Sylvaine Huc-Brandt, Linda Pätzold, Laila Gannoun-Zaki, Ahmed Mohamed Mostafa Abdrabou, Markus Bischoff, Virginie Molle

**Affiliations:** 1 Institute of Medical Microbiology and Hygiene, University of Saarland, D-66421 Homburg, Germany; Mohamed.Elhawy@uks.eu (M.I.E.); Linda.Paetzold@uks.eu (L.P.); Ahmed.Mostafa@uks.eu (A.M.M.A.); 2 Department of Pathology, Faculty of Veterinary Medicine, Zagazig University, Zagazig 44511, Egypt; 3 Laboratory of Pathogen Host Interactions, Université de Montpellier, CNRS, UMR 5235, 34095 Montpellier, France; sylvaine.huc-brandt@umontpellier.fr (S.H.-B.); laila.gannoun@umontpellier.fr (L.G.-Z.); 4 Medical Microbiology and Immunology Department, Faculty of Medicine, Mansoura University, Mansoura 35516, Egypt

**Keywords:** *Staphylococcus aureus*, arginine phosphatase, infection, oxidative response

## Abstract

*Staphylococcus aureus* continues to be a public health threat, especially in hospital settings. Studies aimed at deciphering the molecular and cellular mechanisms that underlie pathogenesis, host adaptation, and virulence are required to develop effective treatment strategies. Numerous host-pathogen interactions were found to be dependent on phosphatases-mediated regulation. This study focused on the analysis of the role of the low-molecular weight phosphatase PtpB, in particular, during infection. Deletion of *ptpB* in *S. aureus* strain SA564 significantly reduced the capacity of the mutant to withstand intracellular killing by THP-1 macrophages. When injected into normoglycemic C57BL/6 mice, the SA564 Δ*ptpB* mutant displayed markedly reduced bacterial loads in liver and kidney tissues in a murine *S. aureus* abscess model when compared to the wild type. We also observed that PtpB phosphatase-activity was sensitive to oxidative stress. Our quantitative transcript analyses revealed that PtpB affects the transcription of various genes involved in oxidative stress adaptation and infectivity. Thus, this study disclosed first insights into the physiological role of PtpB during host interaction allowing us to link phosphatase-dependent regulation to oxidative bacterial stress adaptation during infection.

## 1. Introduction

*Staphylococcus aureus* has emerged as a major human pathogen responsible for hospital and community-associated infections that can involve almost any organ system, including skin and soft tissue infections, necrotizing pneumonia, and infective endocarditis [1]. The increase in multi-resistant variants of this species, coupled with its increasing prevalence as a nosocomial pathogen, is of major concern [2]. The success of *S. aureus* as a pathogen and its ability to cause such a wide range of infections are the result of its large armamentarium of virulence factors that is controlled by a sophisticated network of regulatory molecules [3,4]. *S. aureus* has an exceptional ability to survive under unfavorable conditions, either by adapting to environmental factors or by defending against exogenous stresses [5]. Within the human host, the bacterium can infect and reside in a wide range of tissues, ranging from superficial surfaces like the skin to deeper tissues such as the gastrointestinal tract, heart and bones. In order to achieve this multifaceted lifestyle, *S. aureus* uses complex regulatory networks to sense diverse signals that enable it to adapt to different environments and modulate virulence [6].

To survive in changing environments, bacteria have developed exquisite systems that not only sense these stresses but also trigger appropriate responses, which allow survival and even propagation under these conditions. Basic research of staphylococcal protein regulation is therefore required to decipher molecular and cellular mechanisms that underlie pathogenesis, host adaptation and virulence. Thus, understanding how *S. aureus* regulates its virulence in response to host environments is important to devise effective treatment strategies.

Signal transduction is an essential and universal mechanism that allows all cells—from prokaryotes to eukaryotes—to translate environmental signals into adaptive changes. By this mechanism, extracellular inputs propagate through complex signaling networks whose activity is often regulated by reversible protein phosphorylation [7]. Protein kinases and phosphatases are the regulatory proteins that tend to amplify an external signal, thus making it crucial. Protein phosphorylation is carried out by a multitude of protein kinases that transfer the gamma-phosphate from ATP to specific amino acids on proteins, predominantly to the side chains of serine, threonine, and tyrosine residues [8,9]. An analogous number of protein phosphatases counteract these reactions by catalyzing the dephosphorylation of specific substrate proteins. Based on their sequence, structure, and function, protein phosphatases are grouped into three main classes. Phosphatases acting on phospho-serine/threonine (pSer, pThr) comprise the PPP (phospho-protein phosphatase) and PPM (Mg^2+^/Mn^2+^-dependent protein phosphatase) families, whereas enzymes acting on phospho-tyrosine (pTyr) constitute the protein tyrosine phosphatase (PTP) superfamily [8,10]. In addition, specialized protein phosphatases act on phospho-aspartate, phospho-histidine, and phospho-cysteine residues [11,12,13,14]. Recently, a member of the low-molecular weight protein tyrosine phosphatase (LMW-PTP) family was shown to efficiently target phospho-arginine [15]. Protein arginine phosphorylation represents a novel posttranslational modification (PTM) that alters protein function in vitro and in vivo [16,17]. While bacterial tyrosine phosphatases can be intimately involved in a number of cellular processes, one major theme has become apparent with the involvement of phosphatases as virulence factors [18]. Although a detailed picture is yet unavailable, a role of bacterial protein tyrosine phosphatases during host infection has been identified in different facultative and obligate intracellular pathogens, and the strategies employed by them are currently being elucidated [18,19].

A wealth of information has been gained from studies aimed at deciphering the pathophysiological events during *S. aureus*-macrophage infection [20], but the signaling pathways leading to these adaptations are still poorly understood. Protein phosphatases are suggested to be important regulatory enzymes in pathogenic bacteria, though the upstream signaling of these proteins needs to be further analyzed under specific environmental conditions, which may help in identifying their sensing mechanisms. *S. aureus* has been reported to produce two LMW-PTPs, PtpA and PtpB [21], with the corresponding genes being part of the *S. aureus* core genome [22]. In our previous work, we demonstrated the secretion of the *S. aureus* phosphatase PtpA [23], and its involvement in the process of infection and intracellular survival [24]. Furthermore, while PtpB was initially described as a tyrosine phosphatase [21], recent phosphoproteomic analysis indicated that PtpB functions as an arginine phosphatase [25] that is involved in oxidative stress response, amino acid metabolism, and virulence factor synthesis [26]. Yet, its role during infection remains to be investigated. In this study, we demonstrate that PtpB contributes to the intracellular survival capacity of *S. aureus* within macrophages and participates in the infectivity of this pathogen in a murine *S. aureus* abscess model. In vitro phosphatase activity assays revealed furthermore that PtpB is sensitive to oxidative stress. Additionally, we show that PtpB affects the transcription of various genes involved in oxidative stress adaptation and infectivity.

## 2. Materials and Methods

### 2.1. Bacterial Strains, Media, and Growth Conditions

The bacterial strains and plasmids used in this study are listed in Table 1. All mutant strains and plasmids generated for this study were confirmed by sequencing of the affected region, and by assessing gene transcription by quantitative real-time reverse transcriptase PCR (qRT-PCR). *Escherichia coli* strains were grown at 37 °C in LB medium. *S. aureus* isolates were plated on Tryptic Soy Agar (TSA; BD, Heidelberg, Germany), or grown in Tryptic Soy Broth (TSB; BD) medium at 37 °C and 225 rpm with a culture to flask volume of 1:10. Antibiotics were only used for strain construction and phenotypic selection at the following concentrations: ampicillin, 100 µg/mL; tetracycline, 2.5 µg/mL; erythromycin, 2.5 µg/mL; and chloramphenicol, 10 µg/mL.

### 2.2. Cloning and Expression of PtpA, PtpB and SecA Spot-Tagged Proteins in S. aureus

The *ptpA*, *ptpB*, and *secA* genes were amplified by PCR using *S. aureus* Newman chromosomal DNA as a template, and the Spot fragment was amplified by PCR using the pSpot2 vector as a template (Chromotek, Planegg, Germany) with the primers listed in Table S1. The *ptpA*, *ptpB*, and *secA* plasmids were constructed using NEB Gibson Assembly kit (New England Biolabs, Ipswich, MA, USA). The *ptpA, ptpB* and *secA* purified PCR products were fused to the Spot-tag at the *C*-terminus and Gibson cloned into the KpnI/EcoRI digested pRMC2 vector [31], thus generating pRMC2_PtpA-Spot, pRMC2_PtpB-Spot and pRMC2_SecA-Spot, respectively. The plasmids were propagated in *E. coli* IM08B [28] and electroporated into *S. aureus* strain SA564 [27].

### 2.3. Cloning, Expression and Purification of Recombinant PtpB Proteins.

The *ptpB* derivatives plasmids were constructed using NEB Gibson Assembly kit (New England Biolabs). The *ptpB* gene was amplified by PCR using *S. aureus* Newman chromosomal DNA as a template with the primers listed in Table S1. The *ptpB* fragment was fused to Histidine Affinity Tag (HAT) [32] at the *C*-terminus and Gibson cloned into the NcoI/BamHI digested pET19b vector (Novagen, Madison, WI, USA), thus generating pET19b_PtpB. PtpB_T11I and PtpB_D111A derivatives harboring threonine to isoleucine or aspartic acid to alanine substitutions, respectively, were generated by Gibson assembly (Table S1). Transformed *E. coli* BL21 Star cells were grown at 16 °C in LB medium containing 1 mg/mL of glucose and 100 µg/mL of ampicillin, and protein synthesis was induced with 0.5 mM IPTG overnight. Bacteria were disrupted in a French pressure cell and centrifuged at 14,000 rpm for 25 min. Purifications of the HAT-tagged recombinants were performed using TALON^®^ metal affinity resins (Clontech, Mountain View, CA, USA) accordingly to the manufacturer’s instructions and eluted in 200 mM imidazole, 50 mM Tris-HCl [pH 7.4], 100 mM NaCl, and 10% [vol/vol] glycerol, to be stored at −20 °C.

### 2.4. Construction of the S. aureus ptpB Deletion and Cis-Complementation Strains

For the *S. aureus ptpB* deletion mutants, 0.95- and 1.1-kb fragments (nucleotides 2236272-2237225 and 2234785-2235883 of GenBank accession no. AP009351.1, respectively), containing the flanking regions of the *ptpB* open reading frame (ORF), were amplified by PCR from chromosomal DNA of *S. aureus* strain Newman using primer pairs MBH-510/MBH-511 and MBH-512/MBH-513, respectively (Table S1). The PCR products were digested with KpnI/XhoI and EcoRI/SacI, respectively, and cloned together with an XhoI/EcoRI digested lox66-*erm*(B)-lox71 fragment into suicide vector pBT [30] to generate plasmid pBT *ptpB* KO. Plasmid pBT *ptpB* KO was propagated in *E. coli* strain IM08B [28] and subsequently electroporated directly into *S. aureus* strain Newman to obtain strain Newman Δ*ptpB*, in which nucleotides 17 to 405 of the 420-bp spanning *ptpB* open reading frame were replaced by the lox66-*erm*(B)-lox71 cassette by allelic replacement. The deletion of *ptpB* in Newman Δ*ptpB* was confirmed by sequencing, and the strain was then used as a donor for transducing the lox66-*erm*(B)-lox71 tagged *ptpB* deletion into *S. aureus* strain SA564 [27].

For the *cis*-complementation of the Δ*ptpB*-*erm*(B) mutation in SA564 Δ*ptpB*, a 2.4-kb fragment (nucleotides 2234785-2237225 of GenBank accession no. AP009351.1) covering the C-terminal region of ORF NWMN_2020, *ptpB*, the annotated terminator region of the *NWMN-2020-ptpB* operon, and the N-terminal region of *glyA* was amplified by primers MBH-510/MBH-513 (Table S1), digested with KpnI/SacI, and cloned into KpnI/SacI-predigested plasmid pBASE6 [29] to generate plasmid pBASE6 *ptpB* comp. The plasmid was propagated in *E. coli* IM08B and electroporated into *S. aureus* strain SA564 Δ*ptpB*. Replacement of the *erm*(B)-tagged *ptpB* deletion by the functional *NWMN_2020*-*ptpB* locus was done as previously described [33].

### 2.5. Murine Abscess Model

Animal experiments were performed with approval of the local State Review Board of Saarland, Germany, and conducted following the national and European guidelines for the ethical and human treatment of animals. Preparation of the bacterial inoculum and infection of the animals were carried out as described [34], with minor modifications. Briefly, 100 µL bacterial suspensions containing ~10^7^ colony forming units (CFU) were administered intravenously by retro-orbital injection into female, 8- to 10-week-old C57BL/6N mice (Charles River, Sulzfeld, Germany) that were anesthetized by isoflurane inhalation (3.5%; Baxter, Unterschleißheim, Germany). Immediately after infection, mice were treated with a dose of carprofen (5 mg/kg; Zoetis, Berlin, Germany), and at four days post infection, mice were sacrificed, and livers and kidneys were removed. The organs were weight adjusted and homogenized in PBS (Thermo Fisher, Dreieich, Germany), and serial dilutions of the homogenates were plated on blood agar plates to enumerate the CFU rates in the organs.

### 2.6. Macrophage Culture and Infection

Cells of the human leukemia monocytic cell line THP-1 [35] were obtained from the German Collection of Microorganisms and Cell Cultures (DSMZ no. ACC 16). THP-1 cells were cultured and differentiated into macrophages as described in [36]. For macrophage infection, *S. aureus* cells were grown to the mid-exponential growth phase (i.e., 2 h) in TSB medium. The bacteria were collected by centrifugation at 4,000 g for 10 min and resuspended in PBS. The differentiated THP-1 macrophages (5 × 10^5^ cells/well) were inoculated with *S. aureus* at the multiplicity of infection (MOI) of 20:1 (bacteria to cells) and incubated in 10% fetal calf serum (FCS; PAA, Pasching, Germany)-supplemented RPMI-1640 (Thermo Fisher) at 37 °C and 5% CO_2_ for 1 h. Subsequently, cells were washed with PBS to remove unbound bacteria, and the remaining extracellular bacteria were killed by incubation with 100 µg/mL gentamicin (Merck, Darmstadt, Germany) and 20 µg/mL lysostaphin (Genmedics, Reutlingen, Germany) for 30 min. After gentamicin/lysostaphin treatment, macrophages were rinsed twice with PBS (time point T0), and then further incubated in FCS (10%) and gentamicin (100 µg/mL)-supplemented RPMI-1640 for 45 min (time point T45). Lysis of macrophages and enumeration of intracellular bacteria at time points T0 and T45 was performed as described in [24]. The intracellular survival rate was determined by dividing the CFU rate seen at T45 by the corresponding CFU rates seen at T0.

### 2.7. PtpB Phosphatase Activity Assay

The phosphatase activity of PtpB was assayed in vitro by using a method based on the detection of p-nitrophenol (PNP) formed from p-nitrophenyl phosphate (PNPP) cleavage. Tests were performed in buffer containing 100 mM sodium citrate, 40 mM PNPP, and 3 mM dithiothreitol (DTT) when required. The reaction was initiated by the addition of 0.5 μM PtpB phosphatase or derivatives followed by incubation in 96-well microplates at 25 °C. To test the effect of oxidation, DTT was omitted and H_2_O_2_ (500 µM final concentration) was added, respectively. The phosphatase reaction was monitored at 405 nm (absorption maximum of the generated PNP) using a Spark 20M fluorimeter (Tecan, Lyon, France) microplate reader. All experiments were performed at least in triplicate.

### 2.8. H_2_O_2_ Susceptibility Assays

Minimal inhibitory concentrations of SA564 and its Δ*ptpB* derivative for H_2_O_2_ (Sigma, Saint-Quentin-Fallavier, France) were determined by broth microdilution assays according to CLSI standard M07 [37], however, by using TSB instead of cation-adjusted Mueller-Hinton broth. In a second assay, bacterial cells were cultured in TSB at 37 °C and 225 rpm for 2 h, and subsequently challenged with H_2_O_2_ (50 mM final concentration) for 1 h. At the end of the incubation time, CFU rates of the cultures were determined by plate counting.

### 2.9. NO Susceptibility Assay

SA564 and its isogenic Δ*ptpB* mutant were grown overnight on TSA, and colonies were picked on the next morning and suspended in fresh TSB to a McFarland of 0.5. Cell suspensions were diluted 1:100 in TSB and subsequently mixed 1:1 with TSB supplemented with different concentrations (0, 6.25 and 12.5 µM final concentration) of the NO˙ donor diethylamine NONOate diethylammonium salt (DEA NONOate, Sigma). Growth of the cultures was monitored for 12 h using digital time-lapse microscopy with the oCelloScope instrument (BioSense Solutions, Farum, Denmark) under static conditions at 37 °C and 5% CO_2_. Growth kinetics of the cultures were determined using the Background Corrected Absorption (BCA) algorithm of the UniExplorer software (BioSense Solutions, version 9.0).

### 2.10. pH 5.5 Survival Assay

Exponential growth phase cells (i.e., 2 h) of SA564 and its isogenic Δ*ptpB* mutant were washed with PBS (pH 7.4) and suspended in PBS (pH 5.5) to an OD_600_ of 1. PBS washed cells resuspended in PBS (pH 7.4) served as unchallenged controls. Cell suspensions were cultured for up to 3 h at 37 °C, and OD_600_ values of acid stressed cell suspensions were determined every 30 min. The relative survival rates were determined by dividing the OD_600_ readings of the cell suspensions through the values recorded at time point 0. At 1 h post inoculation, aliquots of acid stress challenged cell suspensions and controls were removed, and CFU rates of the cultures were determined by plate counting.

### 2.11. Measurement of Gene Expression by qRT-PCR

RNA isolation, cDNA synthesis and qRT-PCR were carried out as previously described [38], using the primer pairs listed in Table S1. Transcripts were quantified in reference to the transcription of gyrase B (in copies per copy of *gyrB*).

### 2.12. Statistical analyses

The statistical significance of changes between groups was assessed by the Mann-Whitney *U* test for experiments containing ≥ 4 biological replicates using the GraphPad Prism 6.01 software package (GraphPad, San Diego, CA, USA). *p* values < 0.05 were considered statistically significant.

## 3. Results and Discussion

### 3.1. PtpB Does Not Affect the In Vitro Growth of S. aureus SA564 but Promotes Survival in Macrophages

**Figure 1:**
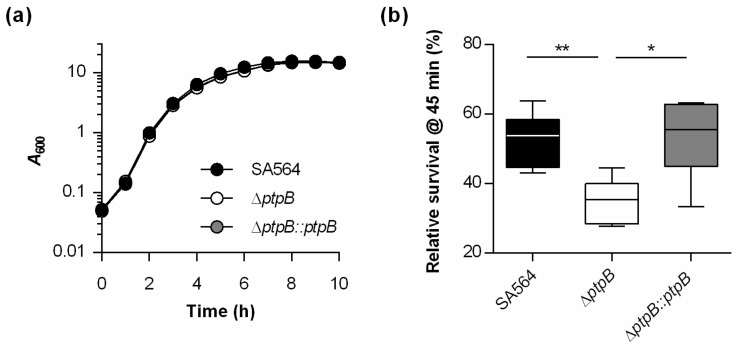
PtpB does not affect growth of *S. aureus* SA564 in TSB but promotes survival in macrophages. (**a**) Growth kinetics of *S. aureus* strains SA564 (black symbols), SA564 Δ*ptpB* (white symbols), and SA564 Δ*ptpB::ptpB* (gray symbols) in TSB. Cells were cultured at 37 °C and 225 rpm at a culture to flask volume of 1:10. Data represent the mean *A*_600_ readings ± SD at the time points indicated (*n* = 3). (**b**) *S. aureus* short-term survival in infected macrophages. Survival rates are given in relationship to the intracellular bacterial cell numbers seen immediately after the gentamicin/lysostaphin treatment, which was set to 100%. The data are presented as box and whisker plot showing the interquartile range (25–75%, box), the median (horizontal line) and the standard deviation (bars) of six independent experiments. * *p* < 0.05; ** *p* < 0.01 (Mann-Whitney *U* test).

Earlier work demonstrated that *S. aureus* survives readily in macrophages [39]. Given the impact of Ptp homologues on survival within macrophages [24,40], we wondered whether PtpB might fulfill similar functions in *S. aureus*. For this purpose, a *ptpB* deletion mutant in *S. aureus* strain SA564, a clinical isolate of clonal complex 5 (CC5), was generated (SA564 Δ*ptpB*), as well as the *cis*-complementation derivative SA564 Δ*ptpB::ptpB*. In vitro growth curves performed with the wild type, the mutant and the complemented strain demonstrated that the deletion of *ptpB* in *S. aureus* SA564 did not markedly affect the bacterial growth in TSB (Figure 1a), which is in line with earlier observations made with *S. aureus ptpB* mutants cultured in full media [26,41]. Next, survival rates of *S. aureus* SA564, the Δ*ptpB* mutant, and the *cis*-complemented strain were determined within THP-1 macrophages at 45 min post Gentamicin treatment (pGt). Already after this short period, a significantly smaller proportion of intracellular surviving cells were observed in THP-1 cells infected with the Δ*ptpB* mutant. *Cis*-complementation of the *ptpB* mutant with a functional *ptpB* locus reverted the intracellular survival rates to levels comparable to the wild type strain (Figure 1b). These data suggest that PtpB is involved in the intracellular survival capacity of *S. aureus* within macrophages.

### 3.2. PtpB Contributes to Infectivity of S. aureus in a Murine Abscess Model

**Figure 2:**
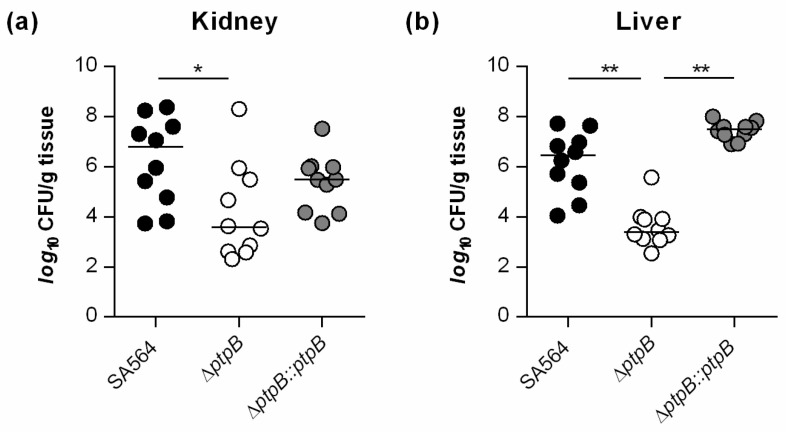
PtpB contributes to infectivity of *S. aureus* SA564 in a murine abscess model. C57BL/6N mice were infected via retroorbital injection with 1 × 10^7^ cells of *S. aureus* strain SA564 (black symbols), SA564 Δ*ptpB* (white symbols), and the *cis*-complemented derivative SA564 Δ*ptpB::ptpB* (gray symbols), respectively (*n* = 10 per group). Mice were euthanized 4 days post-infection, livers and kidneys were removed and homogenized in PBS, and serial dilutions of the homogenates plated on sheep blood agar plates to determine the bacterial loads in kidney (**a**) and liver (**b**) organs. Each symbol represents an individual mouse. Horizontal bars indicate the median of all observations. * *p* < 0.05; ** *p* < 0.01 (Mann-Whitney *U* test).

As PtpB is involved in the survival capacity of *S. aureus* within macrophages (Figure 1b), we wondered whether and how PtpB might affect the infectivity of the bacterium in vivo. To address this, we assessed the ability of the strain triplet SA564/SA564 Δ*ptpB*/SA564 Δ*ptpB::ptpB* to cause disease in a murine abscess model [34]. Coherent with our intramacrophage survival results (Figure 1b), a significant decrease (about 3-*log*) in the bacterial loads in liver and kidney was detected in mice infected with the Δ*ptpB* mutant when compared to mice challenged with the wild type strain (Figure 2). 

Mice infected with the *cis*-complemented derivative SA564 Δ*ptpB::ptpB* displayed comparable bacterial loads in liver as wild type infected mice. In kidneys, SA564 Δ*ptpB::ptpB* infected mice produced CFU rates that were about 1-*log* lower than the CFU rates determined in wild type infected mice (Figure 2a). However, SA564 Δ*ptpB::ptpB* infected mice displayed about 100-fold higher bacterial loads in kidneys than the Δ*ptpB* mutant challenged mice, indicating that the decreases in bacterial loads seen in livers and kidneys of Δ*ptpB* mutant challenged mice were due to the deletion of *ptpB* and not caused by polar mutations. Taken together, these findings suggest that PtpB is a major contributor to abscess formation of *S. aureus* in mice. Remarkably, inactivation of *ptpB* in *S. aureus* decreased abscess formation in both, liver and kidneys, which is not the rule for other mutations affecting liver and renal abscess formation of *S. aureus*, such as *ccpA*, *nor*, *nos*, and *sea* that altered the abscess formation only in one of the two organs upon infection via the blood system [42,43,44].

### 3.3. PtpB Is Not Secreted by S. aureus during In Vitro Growth and Upon Ingestion by Macrophages

**Figure 3:**
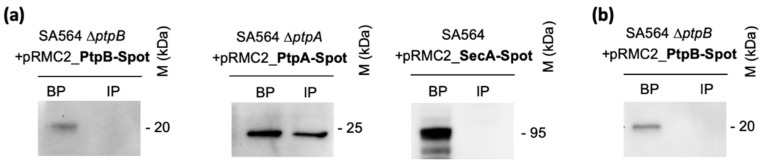
PtpB is not secreted by *S. aureus*. (**a**) Cultures were induced with 0,2 µg/mL of anhydrotetracycline for 2 h and supernatants from *S. aureus* SA564 ∆*ptpB* + pRMC2_PtpB-Spot, SA564 ∆*ptpA* + pRMC2_PtpA-Spot (positive secretion control), and SA564 + pRMC2_SecA-Spot (negative secretion control) were filtered and immunoprecipitated with anti-Spot magnetic beads, whereas bacterial pellets were resuspended and lysed in PBS with protease inhibitor cocktail, lysostaphin, and DNAaseI. Immunoprecipitated proteins (IP) from supernatants and bacterial pellets (BP) were resolved on SDS-PAGEs, transferred to PVDF membranes, and subjected to Western-blot analyses using an anti-Spot antibody as primary antibody (Chromotek) and a HRP-coupled goat-anti-lama antibody as secondary antibody (Bethyl). Data are representative of three independent experiments. (**b**) RAW 264.7 macrophages (5 × 10^5^ cells/mL) were incubated in presence of anhydrotetracycline (0.1 µg/mL) to induce PtpB expression after phagosomal uptake of the bacteria for 2 h with *S. aureus* SA564 ∆*ptpB* + pRMC2_PtpB-Spot at a MOI of 20, and non-phagocytosed bacteria were subsequently removed by gentamicin/lysostaphin treatment. At 3 h pGt, infected macrophages were lysed in 0.1%Triton X-100, and centrifuged at 14 000 g. The obtained supernatants corresponding to macrophage lysates were immunoprecipitated with anti-Spot magnetic beads (Chromotek), whereas the pellets containing intracellular bacteria were resuspended in an equal amount of lysis buffer. Immunoprecipitated proteins and bacterial pellets were resolved on SDS-PAGE and detected with an anti-Spot antibody as described in (**a**). Data are representative of three independent experiments (M kDa: molecular markers).

Various pathogenic bacteria use the secretion of bacterial signaling proteins into target host cells to modulate the phosphorylation status of host signaling networks [45,46,47]. We have recently shown that *S. aureus* PtpA is secreted during in vitro growth or within macrophages [23,24]. Additionally, PtpA has been identified in the secretome of this bacterium [48], despite of the fact that PtpA does not exhibit a general export pathway signal sequence at its *N*-terminus. In order to determine whether PtpB might be secreted by *S. aureus* and interact directly with signaling networks of the host, we created a *C*-terminal Spot-tag *ptpB* translational fusion construct and introduced this construct in *trans* into the SA564 Δ*ptpB* derivative to avoid the expression of endogenous PtpB, generating strain SA564 Δ*ptpB* + pRMC2_PtpB-Spot. The plasmid is expected to drive high levels of expression of a PtpB-Spot fusion protein upon anhydrotetracycline induction [31]. Cultures expressing the PtpB-Spot fusion protein were induced and grown in liquid media for only two hours in order to minimize cell death and lysis. Presence of PtpB-Spot in supernatants and bacterial cells was determined by Western-blot analyses using anti-Spot antibodies. SA564 derivatives expressing Spot-tagged versions of PtpA and SecA served as positive and negative secretion control, respectively [24,49] (Figure 3). 

Cultures expressing the PtpB-Spot fusion construct allowed the identification of the fusion protein within the bacterial pellet but did not indicate the presence of PtpB-Spot in immunoprecipitated culture supernatants (Figure 3a). As a positive secreted control, we used our recently constructed *C*-terminal Spot-tag translational fusion to the *S. aureus ptpA* gene in the pRMC2 vector [24], and introduced this construct into strain SA564 Δ*ptpA*. A *C*-terminal Spot-tag translational fusion to the *S. aureus secA* gene cloned into pRMC2 served as negative control to monitor the potential release of cytosolic proteins by cell lysis. In line with our earlier observations [23,24], we observed clear signals for PtpA-Spot in the bacterial pellets and in immunoprecipitated supernatant fractions, while SecA-Spot was exclusively found in bacterial pellets (Figure 3a), indicating that PtpA is secreted by *S. aureus* during exponential growth, while PtpB is kept within the bacterial cell under these growth conditions. The lack of a SecA-Spot signal in the IP culture supernatant fractions indicates furthermore a negligible release of intracellular proteins via cell lysis under the growth conditions used in this assay, supporting the idea that PtpA is actively secreted by *S. aureus* into the extracellular milieu by a yet unidentified mechanism [24]. Moreover, to test whether PtpB might be secreted by *S. aureus* under infection mimicking conditions, we next infected THP-1 macrophages with the SA564 Δ*ptpB* + pRMC2_PtpB-Spot derivative, induced the expression of PtpB-Spot by addition of anhydrotetracycline to the infected macrophages, and tested the presence of PtpB-Spot in lysed macrophage supernatants and intracellular persisting bacteria three hours post infection. Similar to our observations made with the in vitro cultured cells (Figure 3a), we failed to detect a PtpB-Spot signal in macrophage lysates upon cell infection, while a clear signal could be detected in bacterial cell pellets (Figure 3b), demonstrating that PtpB-Spot was produced by THP-1 ingested *S. aureus* cells. Taken together, these findings indicate that PtpB is not actively secreted by *S. aureus* to modulate host cell signaling, suggesting that the phospho-arginine phosphatase affects the macrophage survival capacity and infectivity of *S. aureus* via intracellular pathways.

### 3.4. S. aureus PtpB Phosphatase Activity Depends on a Highly Specific Threonine Residue in Its Catalytic Loop

**Figure 4:**
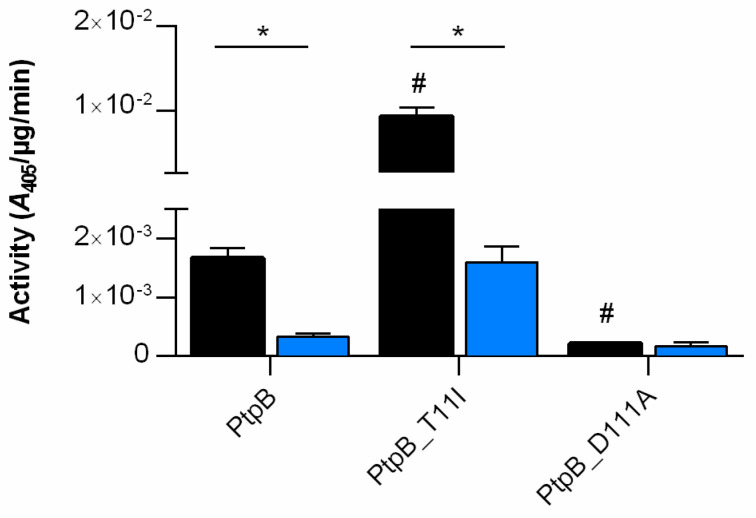
*S. aureus* PtpB phosphatase activity. Recombinant expressed versions of PtpB, PtpB_T11I, and PtpB_D111A were used to test their phosphatase activity on PNPP in presence (black bars) and absence of DTT (blue bars), respectively. Data are presented as mean + SD of four biological replicates. *^,#^*p* < 0.05 (^#^ Mann-Whitney *U* test between PtpB and its derivatives in presence of DTT; * Mann-Whitney *U* test between + DTT and –DTT samples).

PtpB from *S. aureus* was originally characterized in vitro as an acid low-molecular-mass phosphotyrosine protein phosphatases [21,50], although recent in vitro studies reported substrate specific activity of PtpB to release inorganic phosphate from arginine phosphate containing substrates [25,26]. However, the phosphotyrosine phosphatase activity of *S. aureus* PtpB was confirmed by structural motifs typical for LMW-PTPases, which are highly conserved among different staphylococcal strains and even species, and known to play pivotal roles in the catalytic cleavage mechanism [50]. All LMW-PTPs have a specifically shaped binding pocket to distinguish pTyr from other phosphorylated residues. Given this conserved architecture, it is surprising that an annotated LMW-PTP is able to target a different phospho-residue, but this was firstly reported for the LMW-PTP YwlE from *Bacillus subtilis*, which possesses a highly specific phospho-arginine phosphatase activity [15]. YwlE belongs to the LMW-PTP family which contains a conserved active site signature motif, C(X)4CR(S/T) (Supplementary Figure S1), corresponding to the active-site loop (P-loop) region that comprises a conserved cysteine and arginine residue. This P-loop is critical for binding the phosphate group of the incoming substrate and subsequently, to form a transient enzyme-substrate phosphothioester adduct [51]. Overall, YwlE adopts the typical LMW-PTP fold consisting of four β-strands forming a central, highly twisted parallel β-sheet that is flanked by α-helices H1, H2, H5, and H6 on one side, and H3 and H4 on the other side (Fuhrmann et al., 2013a). The P-loop encompassing the C7XXXXXR13 motif connects strand S1 and a helix H1 and constitutes the base of the active-site pocket. As a result, residues Cys7 and Asp118 are properly arranged to dephosphorylate the incoming substrate in a concerted reaction [52]. Although all LMW-PTPs exhibit a similar active-site architecture, in which residues lining the substrate-binding cleft are particularly well conserved, structural comparison of YwlE with the related *S. aureus* PtpA and PtpB phosphatase revealed a remarkable difference regarding position 5 within the CXXXX5XR P-loop (the PX5 residue: Thr in YwlE and PtpB; Ile in PtpA) (Supplementary Figure S1). Furhmann et al. [15] demonstrated already that position PX5 of the CXXXX5XR P loop is critical to direct substrate selectivity either to pArg (PX5 = Thr) or pTyr (PX5 = Ile), as a Thr to Ile exchange increase the pTyr phosphatase activity of YwlE, while reducing its pArg phosphatase activity. We hypothesized that the *S. aureus* PtpB specificity towards pArg is mainly driven by the Thr residue at position PX5, as has been seen with its orthologue YwlE [15]. To assess the role of the critical residues, Thr11 and Asp111, on PtpB phosphatase activity, we constructed single mutants where Thr11 and Asp111 (the catalytically active Asp residue of the conserved D-P-Y triad) were mutated to isoleucine and alanine, respectively. Proteins were overexpressed in *E. coli* and purified as recombinant proteins fused to a HAT-tag. The purified tagged PtpB protein derivatives were then assayed for phosphatase activity in presence of the universal phosphate donor PNPP and the reducing agent DTT (Figure 4, black bars).

The D111A exchange in PtpB strongly decreased the PNPP hydrolysis capacity by a factor of ~7-fold, in line with earlier findings indicating that this Asp residue acts as the general acid catalyst in the transition stage for the phosphate group release [50]. Mutating residue Thr11, on the other hand, had an opposing effect. The T11I exchange in PtpB resulted in a significant increase in PNPP hydrolysis (~6-fold). Therefore, substitution of a single hydroxyl group (threonine) by an ethyl group (isoleucine) led to a drastic change in the phosphatase activity of PtpB as previously described for YwlE from *B. subtilis* [15].

The C(X)4 CR(S/T) motif possessed by LMW-PTPs is crucial for catalysis and redox regulation of their activity. In fact, the dephosphorylation mechanism occurs in a two-step process and involves the formation of a covalent phosphothioester reaction intermediate that is generated by nucleophilic attack of the active site cysteine on the incoming phosphoarginine residue. Studies with other LMW-PTPs suggest that the active site cysteine exists as the negatively charged thiolate anion at physiological pH [53]. In this form, the cysteine residue acts as a strong nucleophile. However, in the latter state, the cysteine residue is also particularly vulnerable to oxidation via reactive oxygen species (e.g., hydrogen peroxide, H_2_O_2_). To further explore the redox-based putative regulation of PtpB, we performed phosphatase assays with our PtpB derivatives in absence of DTT and in presence of H_2_O_2_, respectively. The results of these studies revealed that both the wild-type PtpB and the T11I mutant activities were decreased in absence of the reducing agent by a factor > 5, while the PNPP hydrolase activity of the D111A mutant was not markedly changed (Figure 4, blue bars). No PNPP hydrolase activities were detectable for all three PtpB derivatives in presence of H_2_O_2_ (data not shown), in line with observations obtained with other LMW-PTPs [54,55,56]. Thus, it can be inferred that the redox status of the cell is likely to affect PtpB activity in *S. aureus*.

### 3.5. PtpB Affects the Capacity of S. aureus to Adapt to Oxidative Stress

**Figure 5:**
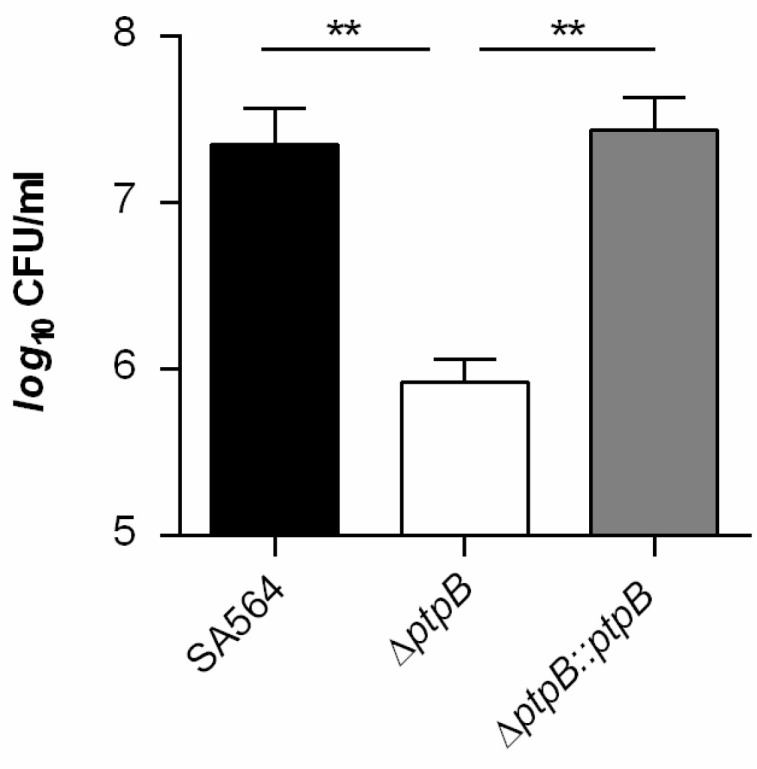
Effect of a *ptpB* deletion on the H_2_O_2_ resistance of *S. aureus* SA564. Cells of *S. aureus* strain SA564, its isogenic Δ*ptpB* mutant, and the *cis*-complemented derivative were cultured in TSB at 37 °C and 225 rpm for 2 h, challenged with 50 mM H_2_O_2_, and CFU rates of the cultures determined at 1 h post H_2_O_2_ challenge. The data presented are the mean + SD of five biological experiments. ** *p* < 0.01 (Mann-Whitney *U* test).

*S. aureus* cells phagocytosed by macrophages are likely to end up in phagolysosomes in which the bacterial cells are challenged among others by reactive oxygen species (ROS) and low pH [57]. Based on our observations that *S. aureus* cells lacking a functional *ptpB* locus have a reduced capacity to survive within THP-1 macrophages (Figure 1b) and that PtpB activity is modulated by oxidative treatment (Figure 4), we wondered whether PtpB might affect the ability of *S. aureus* to cope with oxidative stress. To test this hypothesis, we first determined the minimal inhibitory concentration (MIC) of SA564 and its Δ*ptpB* derivative for H_2_O_2_ using a broth microdilution assay. Unexpectedly, cells of the wild type and the *ptpB* deletion mutant displayed an equal MIC of 0.25 mM. However, when grown in TSB for 2 h and subsequently challenged with 50 mM of H_2_O_2_, SA564 and its Δ*ptpB* derivative produced clearly differing CFU rates at 1 h post H_2_O_2_ challenge (Figure 5). 

Treatment of exponential growth phase cultures of the Δ*ptpB* mutant with H_2_O_2_ significantly reduced the number of CFU, when compared to the CFU rates obtained with H_2_O_2_ challenged cultures of the wild type and the *cis*-complemented derivative, respectively, in line with findings made by Junker et al. [26] for *S. aureus* strain COL. These findings suggest that PtpB does not alter the ability of *S. aureus* SA564 to cope with H_2_O_2_ stress *per se*, but does affect the ability of the pathogen to adapt to oxidative stress during growth.

### 3.6. PtpB Promotes the Capacity of S. aureus to Withstand NO˙ Stress.

**Figure 6:**
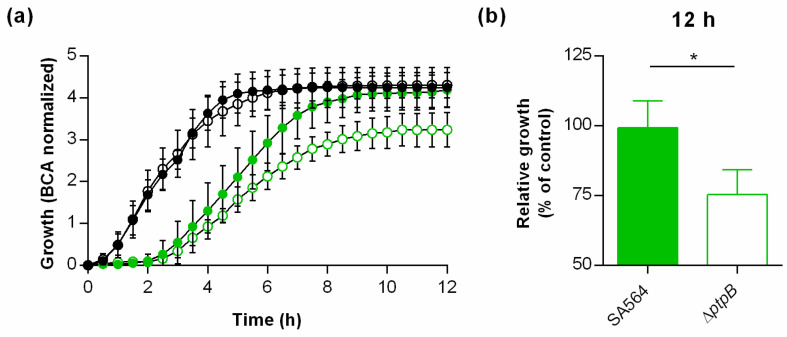
Effect of a *ptpB* deletion on the NO˙ resistance of *S. aureus* SA564. Cells of *S. aureus* strain SA564 (filled symbols) and its isogenic Δ*ptpB* mutant (opened symbols) were cultured in presence and absence of 12.5 µM DEA NONOate in TSB under static conditions at 37 °C and 5% CO_2_ for 12 h. Growth was measured by optical density using the oCelloScope BCA algorithm. (**a**) Growth kinetics of DEA NONOate treated (green symbols) and untreated (black symbols) cell suspensions. The graph represents the average growth values ± SD of four biological experiments. (**b**) Relative growth rates of DEA NONOate treated cells in relation to the growth rates recorded for the untreated controls at 12 h of growth. * *p* < 0.05 (Mann-Whitney *U* test).

In order to test whether PtpB might also affect the capacity of *S. aureus* to cope with NO˙ stress, cells of SA564 and its Δ*ptpB* mutant were challenged with NO˙ donor diethylamine NONOate (DEA NONOate), and growth of the cultures monitored over time (Figure 6). 

Addition of the NO donor at a concentration of 12.5 µM delayed the growth of wild type and Δ*ptpB* mutant cultures for about 2 h, but had only little effect on the growth rate of the wild type after 12 h of growth (Figure 6a,b). In contrast, a clear decrease in growth rates was observed when cells of the Δ*ptpB* mutant were challenged with the NO˙ donor, which reached about 75% of the growth rates seen with the untreated control after 12 h of cultivation (Figure 6b). These findings suggest that PtpB also contributes positively to the capacity of *S. aureus* to recover from nitrosative stress.

### 3.7. PtpB Alters the Transcription of Genes Encoding Factors Involved in the Detoxification of ROS

**Figure 7:**
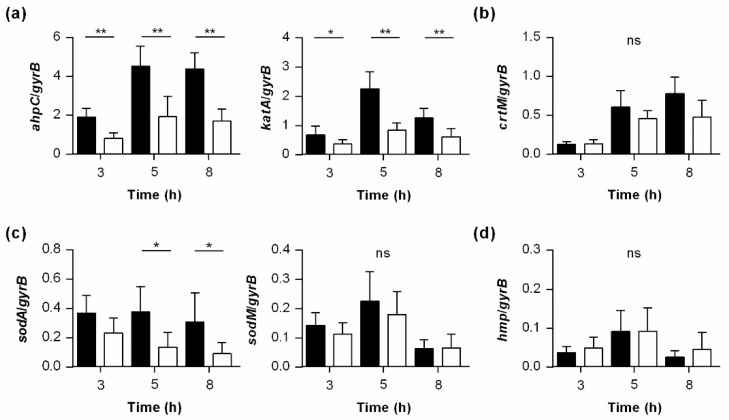
Effect of a *ptpB* deletion on the transcription of genes encoding enzymes involved in the detoxification of ROS in *S. aureus*. Quantitative transcript analyses of *ahpC* and *katA* (**a**), *crtM* (**b**), *sodA* and *sodM* (**c**), and *hmp* (**d**) by qRT-PCR in SA564 (black bars) and SA564 Δ*ptpB* (white bars) cells grown in TSB at 37 °C and 225 rpm to the time points indicated. Transcripts were quantified in reference to the transcription of gyrase B (in copies per copy of *gyrB*). Data are presented as mean + SD of five biological replicates. * *p* < 0.05; ** *p* < 0.01 (Mann-Whitney *U* test between WT and mutant at a given time point).

The adaptive capacity of staphylococcal cells to oxidative stress is of major impact for host infection [58], and our findings show that the lack of *ptpB* decreased the capacity of *S. aureus* to protect itself from H_2_O_2_ and NO˙ induced oxidative stress (Figure 5 and Figure 6). Thus, we wondered whether PtpB might affect the transcription of genes whose products are involved in the detoxification of ROS, specifically of H_2_O_2_. The detoxification of H_2_O_2_ in *S. aureus* is thought to be accomplished mainly by catalase (encoded by *katA*) and the peroxiredoxin alkyl hydroperoxide reductase (encoded by *ahpC*), which convert H_2_O_2_ to water and oxygen [59]. At first, we analyzed the impact of the *ptpB* deletion on *katA* and *ahpC* transcription of *S. aureus* during growth in TSB (Figure 7a). In line with earlier findings [59], we observed a growth phase-dependent transcription of both genes in wild type cells, with a maximum expression in the post-exponential growth phase. Notably, when the transcript rates of *katA* and *ahpC* were determined in cultures of the isogenic Δ*ptpB* mutant along with growth, rather similar growth phase-dependent kinetics were observed. However, when compared in relation to the transcript rates seen for *katA* and *ahpC* in wild type cells at a given time point, Δ*ptpB* mutant cells produced significantly reduced amounts of *katA* and *ahpC* at all three time points analyzed (Figure 7a), suggesting that PtpB is involved in the expression of KatA and AhpC in *S. aureus*.

Another mechanism utilized by *S. aureus* to cope with extracellular H_2_O_2_ stress is the production of the yellow to orange pigment staphyloxanthin [60], which is synthesized from the enzymes coded within the *crtOPQMN* operon [61], and known to be of importance for the resistance of *S. aureus* to phagocytotic killing [60,62,63]. However, we neither observed clear differences in pigment production between the Δ*ptpB* mutant and wild type cells of SA564 during in vitro growth (data not shown), nor in the transcription of *crtM* (Figure 7b), suggesting that PtpB does not affect intramacrophage survival via the modulation of staphyloxanthin biosynthesis.

Encouraged by our findings that PtpB promotes the transcription of *katA* and *ahpC* (Figure 7a), we additionally tested the impact of the *ptpB* deletion on the transcription of the superoxide dismutases encoding genes *sodA* and *sodM* [64,65]. Both gene products are involved in maintaining cell viability during exogenous O_2_^−^ stress by catalyzing the dismutation of O_2_^−^ to oxygen and H_2_O_2_ [58]. While no clear differences in *sodM* transcription between Δ*ptpB* mutant cells and wild type cells were noticed in our qRT-PCR analyses, we observed significantly decreased *sodA* transcript rates in Δ*ptpB* mutant cells for the later time points analyzed (Figure 7c).

Detoxification of nitric oxide in *S. aureus* is mediated mainly by the flavohemoglobin Hmp [66]. To test whether PtpB might also affect the expression of Hmp, we also analyzed the transcription of the respective gene (Figure 7d). Here, no clear differences in *hmp* transcription between Δ*ptpB* mutant cells and wild type cells were noticed, suggesting that PtpB does not modulate the nitrosative stress response of *S. aureus* via transcription of *hmp*, at least under uninduced conditions. Taken together, these data suggest that PtpB is likely to contribute to intramacrophage survival and abscess formation of *S. aureus* via the upregulation of ROS detoxifying enzymes such as AhpC, KatA, and SodA.

### 3.8. PtpB Contributes to the Survival Capacity of S. aureus Under Low pH.

**Figure 8:**
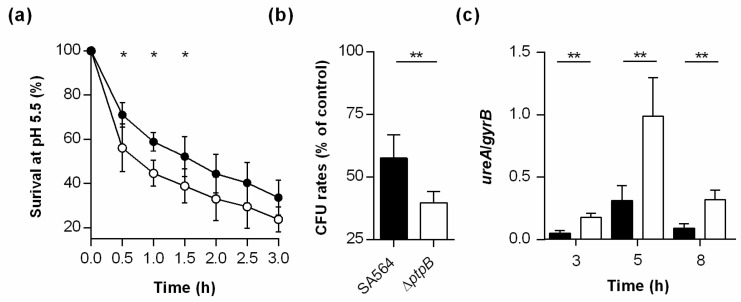
Effect of a *ptpB* deletion on the survival capacity of *S. aureus* at pH 5.5. Exponential growth phase cells of SA564 (black symbols) and its SA564 Δ*ptpB* mutant (white symbols) were inoculated into PBS (pH 5.5) at an OD_600_ of 1 and cultured for 3 h at 37 °C. (**a**) Survival rates over time. Data are presented as mean ± SD of five biological replicates. (**b**) Relative CFU rates of the cultures after 1 h of incubation in PBS at pH 5.5. CFU rates are given in relation to the CFU rates determined in cell suspensions cultured for 1 h in PBS at pH 7.4. Data are presented as mean + SD of five biological replicates. (**c**) Quantitative transcript analyses of *ureA* in *S. aureus* cells grown in TSB at 37 °C and 225 rpm to the time points indicated. Transcripts were quantified in reference to the transcription of gyrase B (in copies per copy of *gyrB*). Data are presented as mean + SD of five biological replicates. * *p* < 0.05; ** *p* < 0.01 (Mann-Whitney *U* test between WT and mutant at a given time point).

*S. aureus* cells ingested by macrophages are challenged among others in the phagolysosome by low pH [67]. To evaluate whether PtpB might also affect the capacity of *S. aureus* to cope with acidic stress, we next tested the survival capacity of wild type and Δ*ptpB* mutant cells in PBS at pH 5.5, an average pH found in phagolysosomes of macrophages loaded with viable *S. aureus* cells [67]. To closer resemble conditions seen in the macrophage phagolysosome, exponential growth phase cells of the wild type and the Δ*ptpB* mutant were challenged with the low pH in PBS and not the culture medium (i.e., TSB). When compared to wild type cells incubated in PBS at pH 5.5, the OD_600_ values of the Δ*ptpB* mutant cell suspensions dropped faster at all time points analyzed (Figure 8a). 

Similarly, cell suspensions of the Δ*ptpB* mutant produced significantly smaller relative CFU rates after 1 h of cultivation than wild type cultures (Figure 8b), suggesting that PtpB contributes to the ability of *S. aureus* to survive under pH conditions encountered by the bacterial cell in the macrophage phagolysosome. As urease was recently reported to represent an essential component of the acid response network of *S. aureus* [68], we next tested whether PtpB might affect the transcription of the corresponding operon, *ureABCEFGD*. Our qRT-PCR analyses revealed that the *ptpB* deletion indeed strongly affected the transcription of *ureA* in SA564 (Figure 8c), however, unlike expected. When cultured in TSB, cells of the Δ*ptpB* mutant produced significantly increased transcript rates of *ureA* than wild type cells at all time points analyzed, suggesting that PtpB represses the transcription of the *ureABCEFGD* operon during in vitro growth. The enhanced level of *ureA* transcripts seen in Δ*ptpB* mutant cells is probably due to a reduced production of the transcriptional regulator CodY, a known repressor of urease gene transcription in gram-positive bacteria [68], as Junker et al. [26] reported significantly lower amounts of CodY in Δ*ptpB* mutant cells when compared to wild type cells. However, if and how a reduction of urease affects the ability of *S. aureus* to survive within the macrophage phagolysosome remains subject to further analysis.

## 4. Conclusions

Phosphatases expressed by pathogenic bacteria can act as key regulators of important microbial processes, especially during environmental adaptation encountered upon host infection [18,19]. Our macrophage survival-, mice infection-, and transcription data suggest a similar role for PtpB in *S. aureus*. Interestingly, *S. aureus* PtpB presents important differences compared to its LMW-PTPs orthologues. In particular, we provide evidence that PtpB phosphatase activity could be influenced by a single Thr residue in its catalytic loop. Our observations that PtpB activity is affected by its redox status suggest a putative role during the oxidative stress response, as has been seen for the PtpB homolog YlwE in bacilli [56,69]. In line with this assumption, we observed a decreased capacity of the *ptpB* deletion mutant to withstand H_2_O_2_, NO˙, and low pH stresses, skills that are probably very important for *S. aureus* to survive inside macrophages [70]. Professional phagocytes such as macrophages express a multifaceted antimicrobial arsenal, which includes but is not limited to the production of reactive oxygen or nitrogen species (ROS, RNS), and antimicrobial peptides in an acidified environment [71]. Nevertheless, *S. aureus* can withstand these mechanisms and conditions [70]. It is thus tempting to speculate that PtpB redox regulation might participate in the adaptation processes initiated by *S. aureus* to survive and replicate within the phagolysosome. However, further work will be needed to decipher the molecular mechanisms utilized by PtpB that allow *S. aureus* to adapt to oxidative stresses encountered in macrophages and during infection.

## Figures and Tables

**Table 1:** Strains and plasmids used in this study.

Strain	Description ^1^	Reference or Source
*S. aureus*		
SA564	*S. aureus* clinical isolate, wild type	[27]
SA564 Δ*ptpB*	SA564 Δ*ptpB::*lox66-*erm*(B)-lox71; Erm^R^	This study
SA564 Δ*ptpB::ptpB*	*cis*-complemented SA564 Δ*ptpB* derivative	This study
*E. coli*		
BL21(DE3)Star	*E. coli* strain allowing a high-level recombinant protein expression. IPTG-inducible T7 RNA polymerase	Invitrogen
IM08B	*E. coli* DC10B derivative harboring *hsdS* of *S. aureus* strain NRS384, Δ*dcm*	[28]
TOP10	*E. coli* derivative ultra-competent cells used for general cloning	Invitrogen
Plasmids		
pBASE6	*E. coli–S. aureus* temperature-sensitive suicide shuttle vector, *secY* counterselection; *bla cat*	[29]
pBASE6 *ptpB* comp	pBASE6 derivative harboring the C-terminal region of NWMN_2020, *ptpB*, and the N-terminal region of *glyA*; *bla*, *cat*	This study
pBT	*S. aureus* suicide plasmid; *tet*(L)	[30]
pBT ptpB KO	pBT derivative harboring the genomic regions flanking *ptpB* and lox66-*erm*(B)-lox71; *tet*(L), *erm*(B)	This study
pET19b	*E. coli* vector for IPTG inducible protein expression; *bla*	Novagen
pET19b_PtpB	pET19b derivative used to express HAT-tagged fusion of *S. aureus* PtpB WT in *E. coli*; *bla*	This study
pET19b_PtpB_D111A	pET19b derivative used to express HAT-tagged fusion of *S. aureus* PtpB_D111A in *E. coli*; *bla*	This study
pET19b_PtpB_T11I	pET19b derivative used to express HAT-tagged fusion of *S. aureus* PtpB_T11I in *E. coli*; *bla*	This study
pRMC2	*E. coli–S. aureus* shuttle vector, Tetracycline-inducible expression; *bla*, *tet*	[31]
pRMC2_PtpA-Spot	pRMC2 derivative used to express C-terminal Spot-tagged fusion of *S. aureus* PtpA; *bla*, *tet*	This study
pRMC2_PtpB-Spot	pRMC2 derivative used to express C-terminal Spot-tagged fusion of *S. aureus* PtpB; *bla*, *tet*	This study
pRMC2_SecA-Spot	pRMC2 derivative used to express C-terminal Spot-tagged fusion of *S. aureus* SecA; *bla*, *tet*	This study

^1^ Erm^R^, erythromycin-resistant.

## Data Availability

The datasets generated during and/or analyzed during the current study are available from the corresponding author on reasonable request.

## Supplementary Materials

The following are available online at https://www.mdpi.com/2073-4409/10/3/645/s1, Table S1: Primers used in this study; Supplementary Figure S1: Alignment of LMW-PTPs.
