# Supplementary material for: The Phosphoarginine Phosphatase PtpB from Staphylococcus aureus Is Involved in Bacterial Stress Adaptation during Infection
Source: Cells. 2021 Mar 14;10(3):645. doi: 10.3390/cells10030645 (PMC8001253; doi:10.3390/cells10030645)
Supplement: Supplementary file 1 [file cells-10-00645-s001.pdf]

Supplementary Material

The phosphoarginine phosphatase PtpB from *Staphylococcus aureus* is involved in bacterial stress adaptation during infection

Mohamed Ibrahem Elhawy [1,3], Sylvaine Huc-Brandt [2], Linda Pätzold [1], Laila Gannoun-Zaki [2], Ahmed Mostafa M. Abdrabou [1,4], Markus Bischoff [1,*], and Virginie Molle [2,*]

[1] Institute of Medical Microbiology and Hygiene, University of Saarland, Homburg/Saar, Germany
[2] Laboratory of Pathogen Host Interactions, Université de Montpellier, CNRS, UMR 5235, Montpellier, France
[3] Department of Pathology, Faculty of Veterinary Medicine, Zagazig University, Zagazig, Egypt.
[4] Medical Microbiology and Immunology Department, Faculty of Medicine, Mansoura University, Mansoura, Egypt.
* Correspondence: V.M. virginie.molle@umontpellier.fr; Tel.: +33 467144725; M.B. markus.bischoff@uks.eu, +49 6841 1623963

Supplementary Table 1: Primers used in this study.

Primer	Direction	Sequence (5'-3') [1]
**Cloning primer**		
MBH510	For.	gtcggTACCAACTGAAACAGTTTATGGAC
MBH511	Rev.	gtgctcgagACGAATAAAATCTTCATAGTTCACATCC
MBH512	For.	GATATTATTTGCTAGTAGGAATTCTG
MBH513	Rev.	gtcgagCTCCATATTCAACGAAATTGTAG
VM11_PtpA_pRMC2	For.	tatGGTACCGGGATATAACTACATAGT
VM11(1)_ PtpA-spot-Ct_Gib	Rev.	CACGAACACGATCTGGCCCTTCTTTCAAATTTGC
VM11(2)_ PtpA-spot-Ct_Gib	For.	GCAAATTTGAAAGAAGGGCCAGATCGTGTTCGTG
VM26_PtpB_pRMC2	For.	TAAGCTTGATGGTACATTATTAAAGGATGTGAACT
VM27_PtpB-spot-Ct_Gib	Rev.	ACGATCTGGTGACCCGCAAATAATATCTTTTAATT
VM28_PtpB-Spot-Ct_Gib	For.	AAAGATATTATTTGCGGGTCACCAGATCGTGTTCG
VM29_Spot-Ct_pRMC2	Rev.	GACGGCCAGTGAATTCTAAGAACTCCAATGTGATA
VM49_SecA_pRMC2	For.	TAAGCTTGATGGTACAACAGTTTTTAGCTAAAGGAGC
VM50_SecA-spot-Ct_Gib	Rev.	ACGATCTGGTGACCCTTTTCCATGGCAATTTTTGAAT
VM51_SecA-spot-Ct_Gib	For.	AATTGCCATGGAAAAGGGTCACCAGATCGTGTTCG
**qRT-PCR primer**		
ahpC	For.	TCCAACTGAATTAGAAGACT
ahpC	Rev.	GAGAATACATTTACGCCTAAT
crtM	For.	ACAGTAGGTGAAGTATTGAC
crtM	Rev.	ATCGTATGTCTGATGTGTTT
gyrB	For.	GACTGATGCCGATGTGGA
gyrB	Rev.	AACGGTGGCTGTGCAATA
hla	For.	AACCCGGTATATGGCAATCAACT
hla	Rev.	CTGCTGCTTTCATAGAGCCATTT
katA	For.	AATGGACAATGTATATTCAAGT
katA	Rev.	ATCAAATGGATTATCTTTATGGT
sodA	For.	ACCAAGATAATCCATTAACTGA
sodA	Rev.	ATTTTAGGTAATAAGCGTGTTC
sodM	For.	CCAAGATAATCCATTAACAGAA
sodM	Rev.	CCAAACATCAAATAGTAAGATTG

[1] Small letters represent nucleotides that do not fit with the target sequence. Restriction sites used for cloning are underlined.

Supplementary Figure 1

```
                                 1       10         20              30        40
PtpB_S_aureus        .......MKILFVCTGNTCRSPLAESIAKEVMP....NH...QFESRGIFAV.NNQGV
YwlE_B_subtilis      .......MDIIFVCTGNTCRSPMAEALFKSIAEREGLNV...NVRSAGVFAS.PNGKA
Wzb_E_coli           .....MFNNILVVCVGNICRSPTAERLLQRYHP....EL...KVESAGLGAL.VGKGA
AmsI_E_amylovora     .....MINSILVVCIGNICRSPTGERLLKAALP....ER...KIASAGLKAM.VGGSA
Etp_E_coli           .MAQLKFNSILVVCTGNICRSPIGERLLRKRLP....GV...KVKSAGVHGL.VKHPA
PtpA_S_aureus        ......MVDVAFVCLGNICRSPMAEAIMRQRLKDRNIHD.I.KVHSRGTGSWNLGEPP
YfkJ_B_subtilis      ......MISVLFVCLGNICRSPMAEAIFRDLAAKKGLEGKI.KADSAGIGGWHIGNPP
PtpA_M_tuberculosis  ...MSDPLHVTFVCTGNICRSPMAEKMFAQQLRHRGLGDAV.RVTSAGTGNWHVGSCA
Acp1_H_sapiens       .MAEQATKSVLFVCLGNICRSPIAEAVFRKLVTDQNISENW.RVDSAATSGYEIGNPP
Ltp1_S_cerevisiae    MTIEKPKISVAFICLGNFCRSPMAEAIFKHEVEKANLENRFNKIDSFGTSNYHVGESP

                             50         60         70         80           90
PtpB_S_aureus        SNYVEDLVEEHHLAET.TLSQQFTEADLK.ADIILTMSYSHKELI...EAHFG.LQNH
YwlE_B_subtilis      TPHAVEALFEKHIALN.HVSSPLTEELMESADLVLAMTHQHKQII...ASQFGRYRDK
Wzb_E_coli           DPTAISVAAEHQLSLEGHCARQISRRLCRNYDLILTMEKRHIERL...CEMAPEMRGK
AmsI_E_amylovora     DETASIVANEHGVSLQDHVAQQLTADMCRDSDLILVMEKKHIDLV...CRINPSVRGK
Etp_E_coli           DATAADVAANHGVSLEGHAGRKLTAEMARNYDLILAMESEHIAQV...TAIAPEVRGK
PtpA_S_aureus        HEGTQKILNKHNIPFDGMISELF..EATDDFDYIVAMDQSNVDNI....KSINP.NLK
YfkJ_B_subtilis      HEGTQEILRREGISFDGMLARQVSEQDLDDFDYIIAMDAENIGSL....RSMAGFKNT
PtpA_M_tuberculosis  DERAAGVLRAHGYPTDHR.AAQVGTEHLAA.DLLVALDRNHARLL....RQLGVEAAR
Acp1_H_sapiens       DYRGQSCMKRHGIPMSHV.ARQITKEDFATFDYILCMDESNLRDLNRKSNQVKTCKAK
Ltp1_S_cerevisiae    DHRTVSICKQHGVKINHK.GKQIKTKHFDEYDYIIGMDESNINNL..KKIQPEGSKAK

                        100           110        120        130
PtpB_S_aureus        VFTLHEY...VKEA.GEVIDPYGGTKEMYVHTYEELVSLILKLKDIIC.........
YwlE_B_subtilis      VFTLKEY...VTGSHGDVLDPFGGSIDIYKQTRDELEELLRQLAKQLKKDRR.....
Wzb_E_coli           VMLFGHW...DNEC..EIPDPYRKSRETFAAVYTLLERSARQWAQALNAEQV.....
AmsI_E_amylovora     TMLFGHW...INQQ..EIADPYKKSRDAFEAVYGVLENAAQKWVNALSR........
Etp_E_coli           TMLFGQW...LEQK..EIPDPYRKSQDAFEHVYGMLERASQEWAKRLSR........
PtpA_S_aureus        GQLFKLLEF.SNMEESDVPDPYYTN..NFEGVYDMVLSSCDNLIDYIVKDANLKEG.
YfkJ_B_subtilis      SHIKRLLDYVEDSDLADVPDPYYTG..NFEEVCQLIKTGCEQLLASIQKEKQL....
PtpA_M_tuberculosis  VRMLRSFDPRSGTHALDVEDPYYGDHSDFEEVFAVIESALPGLHDWVDERLARNGPS
Acp1_H_sapiens       IELLGSY...DPQKQLIIEDPYYGNDSDFETVYQQCVRCCRAFLEKAH.........
Ltp1_S_cerevisiae    VCLFGDWNTNDGTVQTIIEDPWYGDIQDFEYNFKQITYFSKQFLKKEL.........
```

Supplementary Figure 1: Alignment of LMW-PTPs. Sequence alignment of LMW-PTPs from various species (the sequence names consist of the protein name followed by the source organism). Conserved residues are highlighted in red. The characters in red represent the conservative residues. This figure was created using the ESPript server (Robert & Gouet, 2014).
